# S63. WHICH CLINICAL AND COGNITIVE FACTORS ARE RELATED WITH CHANGES IN JUMPING TO CONCLUSIONS IN FIRST-EPISODE PSYCHOSIS?

**DOI:** 10.1093/schbul/sby018.850

**Published:** 2018-04-01

**Authors:** Susana Ochoa, Raquel Lopez-Carrilero, Eva Grasa, Maria Luisa Barrigón, ESther Pousa, Ana Barajas, Esther Lorente, Jordi Cid, Fermin González, Isabel Ruiz, Trinidad Pélaez

**Affiliations:** 1 Parc Sanitari Sant Joan de Deu; 2 Parc de Salut Marc; 3 Servicio de Psiquiatria y Psicologia Clinica; 4 Hospital del Mar; 5 Les Corts; 7 Hospital Clínic Valencia; 8 Institut de Psiquiatria de Girona, University of Jaen; 9 University of Málaga

## Abstract

**Background:**

The data gathering bias of jumping to conclusions (JTC) consists in a tendency to take a decision without sufficient information. There is evidence that suggests that the JTC bias does not improve (So et al., 2010), however other authors suggest that some psychological interventions such as Metacognitive Training have demonstrated that JTC can be reduced (Aghotor et al., 2010; Moritz et al., 2014; Pankowski et al., 2016; Ochoa et al., 2017). Nevertheless, any study has assessed the clinical and cognitive factor that are related with the improvement of this bias in schizophrenia or first episode psychosis.

The aim of the study is to assess which clinical and cognitive factors are related with the improvement of the JTC after a psychological intervention (Meta-Cognitive or psychoeducational group).

**Methods:**

A total of 113 people were assessed with the beads task in two moments: basal and after 3 months. The sample was composed of people with a recent onset of psychosis, recruited from 9 public centers in Spain. Symptoms were assessed with the PANSS and the Psyrats; insight was assessed with the SUMD and the BCIS, and a neuropsychological battery including TMTA and TMTB, digits, WSCT and IQ was used.

**Results:**

A total of 28 (24.8%) patients performed JTC in the basal assessment; of them 18 improved JTC after the interventions and 10 remains performing JTC. People who improved JTC presented higher levels of insight (p=0.032), better neuropsychological functioning in TMTA (p=0.011), Digits (p=0.033) and IQ (p=0.014). Moreover, people who improved JTC presented a tendency to score lower in hallucinations (p=0.097), and better in the WSCT (p=0.065) and TMTB (p=0.076).

**Discussion:**

Some clinical and cognitive characteristics facilitated that people improved JTC bias. People with a better insight and better scores in attention, memory and IQ have more probabilities to improve JTC after a psychological intervention. These variables should be controlled in the interventions with the idea of better address these.

